# Nano-Inclusions Applied in Cement-Matrix Composites: A Review

**DOI:** 10.3390/ma9121015

**Published:** 2016-12-16

**Authors:** Guillermo Bastos, Faustino Patiño-Barbeito, Faustino Patiño-Cambeiro, Julia Armesto

**Affiliations:** 1 Industrial Engineering School, University of Vigo, Rúa Conde de Torrecedeira 86, 36208 Vigo, Spain; inardesign.gbastos@uvigo.es; 2 Centro de Ciências Exatas e Tecnológicas, Centro Universitário Univates, Rua Avelino Tallini 171, Lajeado RS 95900-000, Brazil; faustino.cambeiro@univates.br; 3 Mining Engineering School, University of Vigo, Campus as Lagoas Marcosende, 36310 Vigo, Spain; julia@uvigo.es

**Keywords:** Nanotechnology, graphene, cement matrix, smart structures, functionalization, inclusions, concrete

## Abstract

Research on cement-based materials is trying to exploit the synergies that nanomaterials can provide. This paper describes the findings reported in the last decade on the improvement of these materials regarding, on the one hand, their mechanical performance and, on the other hand, the new properties they provide. These features are mainly based on the electrical and chemical characteristics of nanomaterials, thus allowing cement-based elements to acquire “smart” functions. In this paper, we provide a quantitative approach to the reinforcements achieved to date. The fundamental concepts of nanoscience are introduced and the need of both sophisticated devices to identify nanostructures and techniques to disperse nanomaterials in the cement paste are also highlighted. Promising results have been obtained, but, in order to turn these advances into commercial products, technical, social and standardisation barriers should be overcome. From the results collected, it can be deduced that nanomaterials are able to reduce the consumption of cement because of their reinforcing effect, as well as to convert cement-based products into electric/thermal sensors or crack repairing materials. The main obstacle to foster the implementation of such applications worldwide is the high cost of their synthesis and dispersion techniques, especially for carbon nanotubes and graphene oxide.

## 1. Introduction

The improvement of concrete properties through its interaction with admixtures has been the focus of attention since its emergence as a construction material. Apart from steel reinforcing bars, different embedded admixtures have been added to cement composites to primarily improve their mechanical performance [1]. In more recent times, nanoadmixtures have also been attracting the widespread interest of researchers due to their capability not only to further improve several mechanical properties of cement-based materials, but also to provide new properties that may lead to a wide range of potential applications. These materials include concrete, mortar and cement paste, which are used in structural elements, pavements, and finishing and repairing products [2,3].

In the last two decades, research on the study and manipulation of matter at the nanoscale has been expanding exponentially, supported by the advances achieved in visualising technologies, such as the atomic force microscope, scanning tunnelling microscope and focused ion beam lithography [4]. This expansion is also corroborated by the acceleration in the proliferation of scientific literature published all around the world [5], as a result of a research race between the world powers [6]. However, investments seem to partially neglect the construction sector to date, since few nanotech applications are currently in implementation. The situation is even more critical in the fields of sustainable construction [7,8] and environmental applications [9], despite the demonstrated benefits in water treatment [10], soil and water remediation [11,12], self-cleaning concrete and glass surfaces, photovoltaic coatings [13], or electrochromic windows—which may potentially provide heating, cooling and lighting savings [14].

As far as the construction industry is concerned, it is obvious that this sector faces certain obstacles to the penetration of new materials and technologies. Within this highly fragmented industry, new knowledge is still based on empirical approaches, as construction works are long-term processes which involve high investments. In such conditions, construction companies usually avoid risks that are inherent to research and tend to be reluctant to use materials that are not specifically listed in official construction Codes [7,8]. Consequently, investment efforts involving nanotechnology are mostly focused on higher profit areas, such as electronics, IT (information technology) and health [15,16].

Despite the modest resources allocated to construction research, some recent findings regarding cement matrix reinforced with nanoinclusions point to a noticeable improvement in the mechanical performance and durability of the hardened cement-matrix composite. Additionally, it can be provided with smart features by virtue of its physical characteristics, either through its dispersion into the cement matrix or by being applied as coatings on the cement matrix surface.

**Figure 1:**
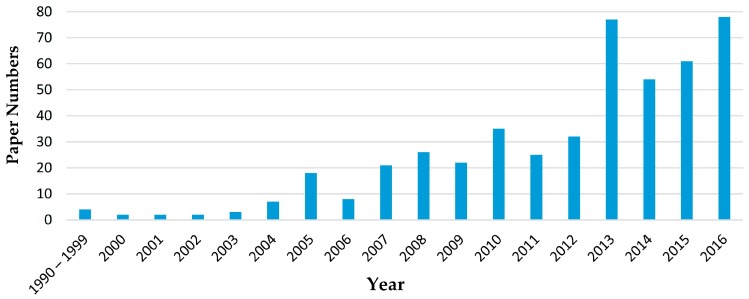
Number of published papers that, according to Scopus, include the terms cement and nanotechnology or nanomaterials in the title, abstract or keywords, limited to the fields of engineering and materials science.

This paper collects the key findings regarding all the aforementioned functionalities derived from the combination of the most studied nanomaterials with the cement matrix. Besides, it gathers the most updated information with regard to the results concerning the strengthening of the cement matrix. In order to illustrate the evolution of this field, a bibliometric study has been conducted (see Figure 1). Although relevant studies were published at the beginning of the 21st century, Figure 1 reveals that the scientific literature has significantly increased in the last decade. Therefore, the authors have mainly focused on the findings reported in this period, paying special attention to the last two years.

At the beginning of the research expansion of nano-modified cement composites, Sobolev et al. published a pioneering two-part review [17,18] on this field. In their work, they paid special attention to nanoparticles and carbon nanotubes (CNTs), as Balaguru et al. also did in their review published in the same year [19]. At that early stage of research, other papers that investigated cement composites containing specific inclusions attracted the attention of the scientific community worldwide; for instance, those focused on nanosilica compared with micro-sized silica fume [20], nanosilica [21,22], nanosilica and nano-Fe_2_O_3_ [23], nanoalumina [24], nanoclay [25], and carbon nanotubes [26,27,28].

More recently, a general description of the applications of nanotechnology to cement was made by Sanchez et al. [29] in 2010. Their work covered the analysis of nanostructures present in the cement matrix, the technological advances that have allowed the characterization of nanomaterials, the effect of carbon nanotubes on the matrix, and the most studied nanoparticles, including references which date primarily from the period 2004–2009. Pacheco-Torgal et al. [30] made a review on the application of nanotechnology to building materials—with a focus on the photocatalytic effect of nanotitania particles—based on different papers mainly published in the period 2003–2010. They also included the currently active topic of the toxicity of nanoparticles. In 2014, the review by Chuah et al. [31], covering mainly the period 2008–2013, incorporated the topic of the fabrication of nano-modified cement composites, focusing on carbon nanotubes and graphene oxide. They analysed their effect on the properties of the fresh cement matrix, the hardened matrix, and the kinetics of hydration. As for the cementitious composites using specific types of nanoinclusions, the following reviews can be highlighted: nanoparticles [32,33], carbon-based nanomaterials [34,35], and CNTs [36,37]. Although more extensive studies based on the collection of previous research have recently been made [38,39,40], some relevant topics have not been covered yet, such as the use of graphene oxide as a reinforcement, the hardening of the surface through the electrochemical migration of nanoparticles, the crack filling carried out by bacterial activity, or the health risks that nanomaterials involve.

In this paper, the authors provide a general overview of the technological peculiarities of nanotechnology when applied to cement-matrix composites and, also, an outline of the state-of-the-art research on mechanical reinforcement from a quantitative approach. In addition, the most remarkable and novelty features achieved are also highlighted. These physical synergies allow cement-based products to become either highly specialised—usually in terms of mechanical performance—or multi-functional—with regard to their electrical, auto-sensing, and thermal behaviour [41]. Moreover, nanomaterials have the potential of leading to considerable savings in terms of materials and resources at each stage in the life cycle of cement products [42].

Therefore, this article is structured as follows. Section 2 introduces nanoscience in the context of cementitious products and, in particular, Section 3 focuses on the techniques required to maximise the interaction between nanomaterials and the cement matrix. The most studied materials, or those with the most promising features because of their synergies with the cement matrix, are described in Section 4. Section 5 collects the main risks that the contact with nanomaterials poses to human health. In Section 6 the findings from the most recent studies are discussed, and, finally, the conclusions are presented in Section 7.

## 2. Concepts of Nanoscience and Nanotechnology Applied to Cement-Based Composites

Given their small size, nanomaterials require advanced technology to be studied, produced, and applied. In order to better understand the properties of nano-modified cement-based materials at the macroscale, it is essential to know the nanostructure of the cement matrix. This knowledge helps to understand the interaction between the matrix and the nanoinclusions, as well as the properties that emerge from their synergy.

Nanoscience is a modern discipline concerned with the novel properties of materials that emerge at the nanometric scale—which is somehow equivalent to the molecular scale. Given the small size of nanoentities, their high specific surface area, the self-assembly characteristic of molecules and the quantum effect, the properties of materials at this scale can vary substantially when compared to higher scaled bulk materials. Thus, for instance, the melting temperature can decrease, and substances can become soluble, transparent, flammable, more reactive, more electrically conductive or catalytic, among other aspects [43]. Therefore, as we move downwards from this scale, the behaviour of the matter becomes far more complex, thus involving the need of an interdisciplinary approach and the confluence of different fields for its study, such as chemistry, physics, engineering, medicine and computing [44].

Nanotechnology studies the manipulation techniques of materials at the molecular level to create large structures. The aim is to exploit the novel and significantly improved properties by gaining control of the structures, while maintaining them stable, in order to integrate these nanostructures at higher scales. Therefore, the obtained composites can be multifunctional, i.e., showing two or more properties, such as greater mechanical performance, different electrical resistance, or self-sensing, self-cleaning and self-healing features [45]. In the scientific literature consulted for this paper, the numeric value adopted for this scale is 1–100 nm, as proposed by the National Nanotechnology Initiative, a programme released by the US Government [46].

In the field of cement matrix, nanoscience studies the structural variables at the micro- and nanoscales in cement-based composites and, by using characterization techniques and molecular modelling, it also analyses their influence on the properties of the composite at the macro-scale. Nanotechnology focuses on the manipulation techniques of these materials with the main goal of improving their performance in a certain way [46]. This enhancement can be applied to three different technical aspects: the behaviour of the fresh cement matrix; the chemical, thermal and mechanical progress during the curing process; and the properties of the hardened composite [47]. To this end, several nanoinclusions can be added to the cement. They basically consist of nano-sized particles, fibres or sheets that are either embedded into the matrix to control the behaviour of the bulk material or grafted onto cement matrix molecules, aggregates or additives in order to modify the interaction between interfaces [29]. They can even be applied as a coating when a small quantity is required or when the desired feature consists in a superficial interaction.

**Figure 2:**
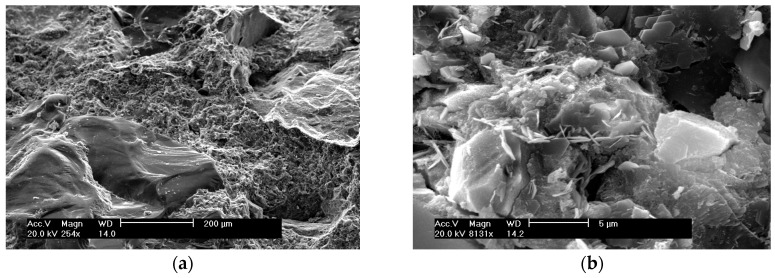
SEM image of concrete with the following composition: ordinary Portland cement CEM II 52.5 (EN 197-1:2000), naphthalene-based superplasticiser, river sand and crushed granite as fine and coarse aggregates, respectively, and a water-to-binder ratio of 0.3 (reproduced from [55]): (**a**) Magnification of 254; and (**b**) Magnification of 8131.

Before covering the benefits provided by nanoinclusions, it must be remarked that the cement matrix is a nano-structured material itself. Since the properties of each scale derive from the structure of the next smaller scale [29], nanoscience has been aimed at revealing the complex relationships between nanostructures and the properties of the bulk material of cement matrices. A micrograph of plain concrete is shown in Figure 2. At the nanoscale, the cement-based composite is a complex structured material, composed of an amorphous phase, nano- to micro-sized crystals, and bound water. To this end, the attention is focused on the binding phase, named calcium–silicate–hydrate (C–S–H) gel, since it is responsible for both the intrinsic cohesion of the cement paste and the adherence of the cement paste to fine and coarse aggregates. Therefore, it also provides the mechanical strength to the composite [48]. In fact, the C–S–H gel has been the subject of intensive research, given its complexity, the increasing range and complexity of admixtures and blending materials, and the wide range of experimental and computational tools that have been applied in recent decades to cementitious materials. Some of these tools include: X-ray diffraction (XRD) [49]; Scanning and Transmission Electron Microscopies (SEM and TEM) [50]; nuclear magnetic resonance and small angle neutron scattering [51,52]; atomic force microscopy [45]; and nanoindentation [53,54].

Papatzani et al. [56] reviewed the models proposed in the period 2000–2014 which describe the relation between the C–S–H nanostructure and the mechanical properties at the macro-scale of the hardened composite. They concluded that modern models were, essentially, an extension of the colloidal or layered models suggested in the 1960s, rather than providing a ground-breaking new approach in relation to nanotechnology and computational advances. Regardless of this aspect, these technologies have facilitated the shift from descriptive to predictive models, have contributed to save research resources, and have paved the way to the production of nano-modified cement matrices with minimum Portland cement content. As for the results of nanotechnology when applied to the study of the C–S–H gel, Raki et al. [57] reviewed the pieces of research that focused on the relation between the C–S–H structure and the mechanical properties of the cement matrix, as well as on the techniques to modify C–S–H composites at the nanoscale.

## 3. Dispersion of Nanoinclusions in the Cement Matrix

When nanoelements are mixed with an aqueous compound, they tend to form agglomerates because of the attractive Van der Waals forces—especially with 1D and 2D morphologies with high aspect ratios. Thus, one of the main obstacles to prepare a cement-matrix composite lies in the difficulty to obtain a mix with a uniformly dispersed inclusion [58]. Techniques to achieve the homogeneous dispersion of nanoadmixtures are often required, and these are classified in four main groups: chemical surface modification, physical surface modification (through surfactants or polymer wrappings), and mechanical methods of ultrasonication and stirring. Other used mechanical methods include ball milling, shear mixing, calendaring and extrusion. Ultrasonication has been commonly used to attain uniform dispersion in the cement matrix: an ultrasonic probe imparts excitation energy to break up the nanotube clusters at the expense of achieving decreased aspect ratios [31].

Parveen et al. [59] and Chuah et al. [31] presented their respective reviews on the dispersion methods of CNTs in cement matrices, a nanomaterial with great potential but with challenging dispersion problems. They also included a review on the mechanical improvements obtained by researchers, which resulted to be highly variable. Hassan et al. [60] analysed how the protocol followed in the preparation of CNT-concrete affects compressive strength, thus confirming such high variability. In addition, they also highlighted the need of providing the exact details involved in the dispersion procedure and of standardising the optimal methods.

In the specialised literature consulted, the concept of “functionalization” is often used as a synonym for “chemical modification”. However, in some cases, “chemical modification” refers to the introduction of weak, non-covalent interactions with relatively unreactive molecules. Although “functionalization” is also used in that context, it usually makes reference to the covalent bonding of reactive functional groups with the nanostructure of the matrix [61]. In spite of the fact that non-covalent modification can be an excellent solution for certain applications, the focus is put on the design of new and more efficient covalent linking techniques. Therefore, functionalization is the most common approach to achieve the satisfying dispersion of CNTs; more specifically by applying acid mixtures, which oxidise CNTs and add carboxylic (–COOH) or hydroxyl groups (–OH), thus increasing the solubility of CNTs in the aqueous matrix. Through this method, CNTs contribute to the rigidisation of the hardened matrix, since the attraction created by the covalent bonds of oxide groups makes them become tightly wrapped by the C–S–H phase. Graphene oxide has properties that are substantially different than those of graphene, given the important changes that the functionalization of graphene sheets implies [59].

Apart from solubility, functionalization can provide graphene and carbon nanotubes with additional properties which are suitable for those applications related to electromechanical behaviour, electrochemical sensing, catalysis, or biocompatibility. For this reason, they have become a subject of intensive research for the fabrication of novel hybrid composites [62,63].

## 4. Nanoinclusions

The most successful nanoinclusions in the cement matrix can be classified into carbon-based and non-carbon-based. Most non-carbon-based inclusions consist of particles, primarily pozzolanic and oxide nanoparticles [64]. Pozzolanic materials reinforce the cement matrix by means of their pore-filler effect and their contribution to the generation of the C–S–H gel. The strengthening effect of the pozzolanic group proportionally increases as their size decreases, given the densification and chemical nature of their reinforcing mechanism [65,66]. Oxide nanoparticles are able to provide conventional mechanical improvements, as well as novelty electrical, thermal, or chemical properties to the matrix, among other aspects. The promising special properties present in nanophysics are still more fascinating in the case of carbon-based nanomaterials, which have an unusual and complicated behaviour at the molecular level [67]. Carbon is the only element that has stable allotropes from the zero to the third dimension. The most studied carbon-based nanoinclusions that are being studied in the cement matrix are primarily CNTs, carbon nanofibres (CNFs), graphene oxide (GO), graphite nanoplatelets (GNPs), and carbon black (CB).

A key factor that governs the properties of nanoelements is their morphology. Therefore, the following fundamental classification has been established: zero-dimensional (nanoparticles), one-dimensional (nanofilaments) and two-dimensional (nanosheets). Apart from the properties studied by nanophysics, the morphology of fibres and sheets allows them to behave as support materials that impede crack growth [68]. This reinforcement effect proportionally increases as their aspect ratio and tensile strength rise [69].

### 4.1. Non-Carbon-Based Nanoinclusions: Nanoparticles

Nanoparticles highly attract the interest of the scientific community because of their wide variety of practical applications, including medicine [70], electronics and advanced ceramics [71]. Non-carbon-based materials are commonly presented as particles, since they do not possess the unique characteristic of carbon: its high flexibility to bond with itself and with other molecules. Therefore, in the last decade, the application of nanoparticles to the cement matrix has become a key object of research. Some of the most studied are: pozzolanic nanosilica (SiO_2_) [72], nano-clay and nano-MK (nanometakaolin); nano-Fe_2_O_3_, nanotitania (TiO_2_), nanoalumina (Al_2_O_3_), and nano-MgO oxide particles; and the nano-CaCO_3_ salt. Nanoparticles act as a pore filler and make the C–S–H net more rigid because of their chemical reactivity. As a result, some common positive effects include: improving the mechanical performance, enhancing the corrosion resistance, reducing the shrinkage and permeability of concrete, and increasing the life span of cement-matrix structures [57,73]. In order to provide a general overview of these elements, the most relevant nanoparticles in cement-based composites are presented in the following sections.

#### 4.1.1. Nanosilica

In the case of pozzolanic admixtures at the micro-scale, the size and orientation of C–H crystals tend to decrease, thus improving the connections of the net [74]. These features, together with the densification and chemical bonding of pozzolans, are proportionally fostered as the scale is reduced. Opposite to carbon nanoallotropes, the efficiency in the manufacture of nanoparticles has greatly improved in recent decades, leading to a substantial cost reduction in the use of volumetric admixtures [75]. This has facilitated the penetration of nanoparticles in construction materials, especially of nanosilica, a compound which belongs to the pozzolanic group. This nanoparticle is the most economical, the most studied and, also, the most consumed in cement worldwide [76]. In Table 1, some of the latest results obtained when reinforcing different cement matrices can be observed.

Gonzalez et al. [84] tested how the increase in the compressive strength and water tightness of concrete when using nanosilica, led to a higher resistance of concrete pavement in cold conditions. To this end, they carried out an evaluation of freeze/thaw cycles and of the consequent scaling response. Scaling occurs when the effect of freezing and thawing cycles creates localised failures or mortar degradation on the surface.

Nanosilica has proven to be helpful in facilitating the use of recycled materials in the cement matrix. For example, Mohammed et al. [85] found an improvement in the compressive strength of concrete when using rubber from waste tires and nanosilica, thus allowing a structural use of concrete with a high rubber content. If a decrease in toughness is not desired, nanosilica helps to maintain this feature within the same values as conventional concrete. Li et al. [86] have used nanosilica and nano-CaCO_3_ to maximise the use of the most consumed recycled material in construction: the recycled aggregate concrete.

Based on its high content of silica, Harbec et al. [87] tested recycled glass nanoparticles with cement paste, obtaining a mortar with a compressive strength and permeability equivalent to that of high performance concrete (HPC) with silica fume (SF). More recently, Aly et al. [88] tested mortar with nanosilica and glass microparticles, concluding that nanosilica seemed to facilitate the use of glass particles as a high-volume cement replacement. The specimen, containing 20% of glass powder and 3% of nanosilica, achieved an increase of 31% in compressive strength and of 55% in flexural strength.

A technique which can be potentially applied in the maintenance of concrete structures is based on the electromechanical migration of species. The extraction of chloride from corroded reinforced concrete has been a well-known technique for years: chloride ions are alienated from rebars by applying an electric field to the concrete element [89]. A number of experiments have achieved a compaction effect on concrete by electromechanically injecting nanoparticles in the concrete surface. Díaz-Peña et al. [90] documented a 1.5–2 mm deep protective film by injecting nanosilica. Fajardo et al. [91] achieved a similar pore-filling effect and protection against carbonation using Si^4+^ ions. Shan et al. [92] worked with a solution of SiO_3_^2−^ ions, which is easier to prepare as they are more stable than nanosilica particles. Applying such substances and subjecting them to an electric field on site structures constitutes a complex task. For this reason, Climent et al. [93] increased the efficiency of the process by means of a graphite-cement paste coating used as an anode. Although it performs at an efficiency of 80% when compared to conventional anodes, it is a low cost and durable anode, adaptable to any surface and less sensitive to the anisotropic electric properties of concrete created by the spatial distribution of rebars.

#### 4.1.2. Nanotitania

**Figure 3:**
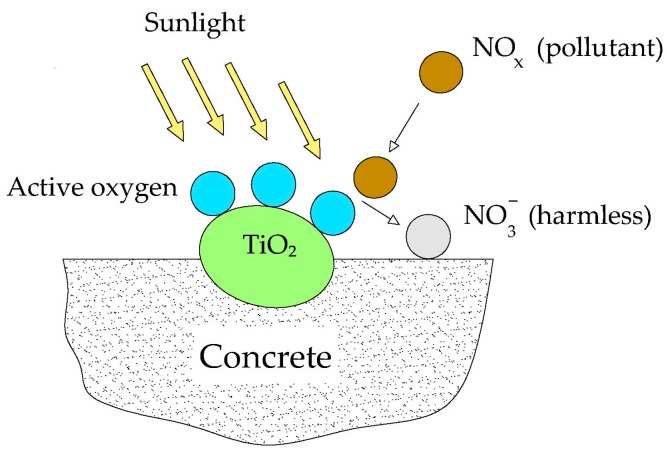
Schematic representation of photocatalytic concrete.

TiO_2_ provides a different and more advanced characteristic: the photocatalytic effect. Nano-TiO_2_ is the most widely used photocatalyst in the field of construction materials and is the second most used nano-oxide particle [94]. It has been successfully applied in the production of self-cleaning concrete that contributes to destroy organic pollutants. The removal of NO*_x_* caused by the photocatalytic reaction is illustrated in Figure 3. Some of the most recent tests on this topic include those carried out by Cerro-Prada et al. [95] and Ganji et al. [96]. Cerro-Prada used Methylene Blue as the organic dye, while Ganji used malachite green—which is severely toxic and difficult to remove from aqueous solutions. Both applied UV radiation. Ganji observed that cement specimens containing nanotitania showed stronger photocatalytic properties compared to those comprising the same amount of pure titania. Cohen et al. [97] documented a more pronounced cleaning effect using nano-TiO_2-*x*_N*_y_* than the one obtained with TiO_2_ nanoparticles, being both activated by UV and visible radiations.

The self-cleaning feature provided by the titanium oxide embedded in the cement matrix has already been put into practice, for example, in the construction of the distinctive Jubilee Church in Rome [98]. However, the term “self-cleaning” must not be taken literally: cleaning these surfaces still requires detergents and water, but in a lower amount than in the case of ordinary materials. Again in Rome, a self-cleaning coating was applied on the Ara Pacis archaeological museum. In this case, the self-cleaning feature mimics the lotus-leaf effect: a hydrophobic effect is achieved by making the surface microscopically rough [99].

Given that this photocatalytic reaction consists in a surface interaction, nanotitania particles are also applied on concrete surfaces as coatings. This is the method studied by Jafari et al. [100], who coated concrete blocks by submerging them in a solution with nanosilica and nanotitania. In their article, they also described how nanosilica fostered the photocatalytic effect of the coating by reducing the size of nanotitania particles. Currently, there are commercial coatings containing nanotitania which are suitable to be applied to concrete [101].

Nanotitania possesses additional features with regard to transparency and, also, as a durability enhancer. Therefore, this admixture, when applied as a coating, can play an interesting role in maintenance, particularly in the case of high cost maintenance structures or valuable cultural heritage buildings. Quagliarini et al. [102] have already tested this aspect in their experiment on travertine—a limestone commonly used in historical and monumental buildings.

Faraldos et al. [103] produced a coating that brings together the photocatalytic and hydrophobic effects. They combined nano-TiO_2_ particles with a siloxane sealant. Ramachandran et al. [104] applied a coating to OPC specimens in order to provide them with hydrophobic and icephobic capabilities. This coating consisted of a water-based siloxane emulsion for hydrophobic modification, polymethylhydroxysilane for the preparation of the hydrophobic agent, and polyvinyl alcohol to be used as a surfactant. PVA fibres, SF, and sand were also added to enhance the icephobic effect. As a result, the coated specimens could repel falling water droplets at −5 °C. In the last few years, other super hydrophobic coatings have been developed and tested—for example nanosilica coatings [13]. These coatings may be applied as an anti-corrosion protection, since water is the cause of most of the pathologies that affect the foundations of buildings.

#### 4.1.3. Most Relevant Lines of Study Using Other Nanoparticles

Nowadays, the comparison or combination of different nanoparticles with other inclusions is one of the most active research topics within this field. In this regard, the study conducted by Mutuk et al. [105], who compared the hardening effect achieved when using different types of nanoparticles, is a remarkable example. They measured, for an ordinary Portland mortar, an improvement in its compressive strength of 16.4%, 15.4%, and 10.5% adding 1% of nanosilica, nano-Al_2_O_3_, and nano-Fe_2_O_3_, respectively. Zhang et al. [106] analysed the pieces of research published between 2004 and 2013 and summarised the improvements achieved in the strengthening effect of the cement matrix with nanosilica, nano-Al_2_O_3_, and nano-TiO_2_.

Liu et al. [107] quantified how the synergy between ground granulated blast-furnace slag (GGBS), a pozzolanic material, and nanosilica could lead to an improvement in compressive strength. They observed that a low content of nanosilica reduced the hydration of the GGBS and, therefore, the porosity of the matrix. An ordinary Portland cement was used to reach a compressive strength of 59.42 MPa after a 28-day curing period when adding 30 wt % of GGBS and 3 wt % of nanosilica. Garg et al. [108] experimented with different combinations of micro- and nanosilica. They concluded that a proportion of 1 wt % of nanosilica and 10% of microsilica led to the maximum improvement in the split tensile strength, apart from providing resistance to the penetration of chloride. Table 1 illustrates the use of nanosilica in combination with different micro- and nanomaterials.

Ismael et al. [109] studied the influence of nanosilica and nano-alumina on the steel-concrete bonding. Although no effect was observed in fibre reinforced concrete, different findings arose in steel reinforced concrete. The bond stress increased approximately by 25% in the case of both nanoparticles when a high dosage of cement was present. When plain rebars were used, nanoalumina was effective in reducing the width of the cracks and the spacing between them.

Beyond the basic properties required for structural elements, Land et al. [110] analysed how the kinetics of cement hydration could be controlled by nanoparticles. Nanosilica resulted to be a cement hardening accelerator, while nanoalumina was identified as a retarder. As Cai et al. [111] demonstrated, nano-CaCO_3_ accelerates the hardening process and decreases the shrinkage, although a high curing humidity of the samples is needed to improve the durability of cement-based composites.

At the nanoscale, another pozzolanic material is also used in the cement matrix: nanomontmorillonite, usually referred as nanoclay. It consists of a three-layered 2-D structure of aluminium inserted between two layers of silicon. Apart from its intrinsic pozzolanic reactivity, nanoclay acts as a plasticiser: it swells to many times its original volume when it absorbs water. Chang et al. [25] studied nanoclay in the cement paste, finding a bigger improvement in the reduction of the permeability than in the compressive strength, as described in Table 1.

Nano-MgO is a newly explored chemical nanocomponent in the application of nanoparticles to the cement matrix. It has been added into mass concrete, as it serves as a shrinkage-compensator, and it is more effective than other expansion agents that often need more water [112]. Regarding the modification in the mechanical performance, Moradpour et al. [83] achieved, after a 28-day curing period and with 1 wt % of nano-MgO, an 80% and a 70% increase in the compressive and flexural strengths, respectively. Jayapalan et al. [113] reported that adequate dosages of nano-TiO_2_ and micro-CaCO_3_ could be useful to control the shrinkage and the environmental impact of cement composites.

### 4.2. Carbon-Based Nanoinclusions

The most studied and recently discovered carbon-based nanoinclusions in the cement-matrix include graphene, CNTs and GO. Graphene can be considered as a two-dimensional material, since it is a sheet of carbon atoms that are individually linked to three other atoms by means of a hybrid sp^2^ bond, thus creating a honeycomb-like net [114]. Until recent years, the two-dimensional graphene was probably the most studied carbon allotrope, but failures in synthetising graphene or any other two-dimensional crystal led to the belief that these elements were not stable in ambient conditions [67]. Once graphene was first isolated by Geim and Novoselov in 2007 [115], an enormous experimental activity began in a great number of lines of research. Graphene is the building block of CNTs, CNFs and GO, which are described in the following sections of this paper. The typical values of their fundamental mechanical properties are gathered in Table 2. As a consequence of the wide range of the production processes applied, a high variability in the values of this property can be observed. The amorphous carbon allotrope known as carbon black is also presented in this paper because it is a cheaper alternative to provide electrical properties to the cement matrix.

Graphite, the raw material for these allotropes, is an abundant resource. However, the synthesis of carbon-based nanomaterials is a demanding challenge that constitutes a huge barrier to the exploitation of their exceptional characteristics. Nevertheless, and given that the technological advantages provided by these materials have already been demonstrated, their demand is greatly increasing, as it illustrates the fact that improved or new synthesis procedures constitute a current trend in research literature. In a very recent survey carried out by Shapira et al. [122] among 65 graphene-based enterprises, it was observed that 60% of them were base material producers. Nonetheless, the electronics, energy, aerospace and automotive fields, as well as the manufacturing industry of composites and coatings, accounted for 71% of the graphene’s expected applications. About 1% of the potential applications of graphene belong to the construction industry [123].

It is at its single-layer form when graphene shows its outstanding physical properties at the highest level. Lee et al. [124] described the state-of-the-art methods for producing such one-atom thick graphene. This study concluded that, in recent works, chemical vapour deposition (CVD) stood out as the most promising procedure to produce pristine graphene at the highest scale, to the detriment of the primitive technique of exfoliating graphite with sticky tape. In order to produce graphene on a large scale, first, graphene oxide is synthetised from graphite and, then, it is reduced through the application of thermal and chemical processes to obtain graphene. The main drawback of this method is the degradation of the mechanical and electrical properties of the graphene obtained [125].

**Figure 4:**
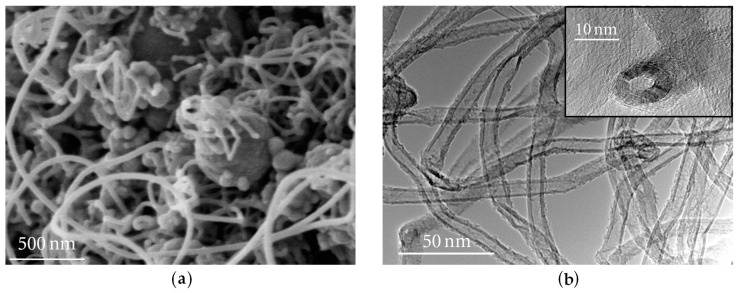
CNTs grown on silica particles with a 100 nm–2 µm diameter at 600 °C: (**a**) Scanning Electron Microscope image; and (**b**) Transmission Electron Microscope image (adapted from [128]).

The most suitable method for synthetising CNTs on a large scale is the CVD, but it creates contaminants which often require costly thermal annealing or chemical treatments to be removed [126]. In their study, Kumar et al. [43] compared the available methods, with their advantages and disadvantages. A more economical alternative for synthetising CNTs was proposed by Sharma et al. [127], which consisted in the use of furnace oil—the cheapest waste from petroleum refineries—as the raw material. They produced CNTs which contained impurities of nano-sized carbon particles and, therefore, the quantity obtained of this nanomaterial was lower than that achieved when using conventional methods. Nonetheless, the nano-reinforced matrix reported an increase of 18% and 34% in the compressive and flexural strengths respectively, and the cost was, approximately, 6% of the price set for pristine CNTs. Figure 4 shows the method for producing CNTs developed by Mudimela et al. [128]—which deals with the problems of improving both the dispersion and the bond with the matrix. They induced the CVD growth of CNTs on silica particles.

The manufacture of GO is commonly based on the Hummers’ method [129] for exfoliating graphite, which is still expensive and involves the generation of toxic and explosive gases [130]. In short, the high cost of these carbon nanoallotropes and the complexity of dispersing them uniformly constitute the main obstacles to the development of cement-matrix applications.

A next step in the evolution of carbon nanoallotropes could lie in the newly born carbyne, which consists of a simple raw of carbon atoms with a sp bond (═C═C═). First synthetisations have already been made, but its properties are still being measured. Kotrechko et al. [131] obtained a tensile strength of 251 GPa at T = 77 k, what would exceed by far graphene’s 130 GPa.

#### 4.2.1. Carbon Nanotubes

CNTs were reported for the first time by Iijima [132,133]. They are usually classified in single-walled (SWCNTs) and multi-walled carbon nanotubes (MWCNTs). A SWCNT is made of a graphene sheet rolled up into a cylinder and closed at both ends by two semispherical caps. Its internal diameter falls within the 0.4–2.5 nm range and its length varies from few microns to several milometers [134]. MWCNTs are made up of more than one graphene cylinder nested into one another. Typical MWCNTs have an inner diameter of 1–3 nm and an outer diameter of 10 nm approx. Their length can be millions of times greater than these measures.

**Figure 5:**
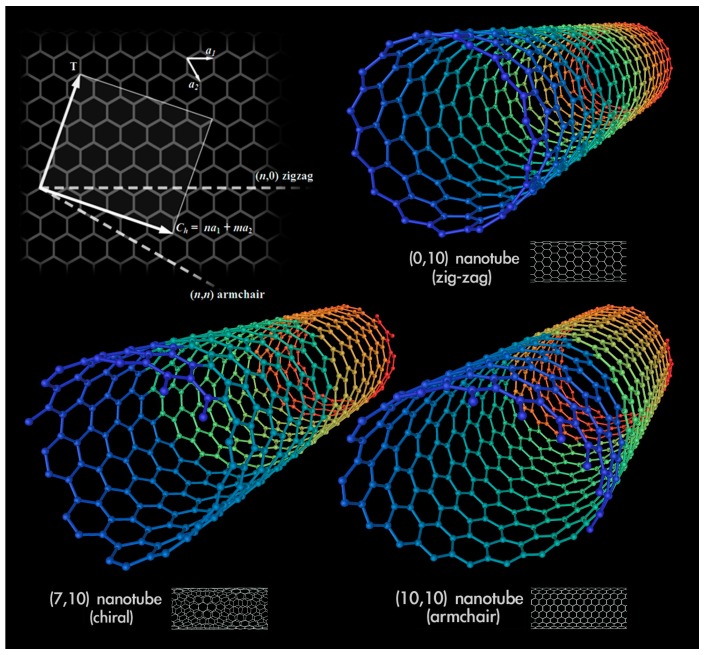
The three types of SWCNTs (adapted from [138]).

Although CNTs have a simple chemical composition, they exhibit one of the most extreme diversity among nanomaterials as far as the structure-property relations are concerned [135,136]. For instance, with regard to the electrical properties, a SWCNT can be a metal, semiconductor or small-gap semiconductor, depending on the orientation of the carbon net on the cylindrical surface (see Figure 5). As for the mechanical behaviour, CNTs are highly resilient, Young’s modulus is roughly 1.2 TPa and their tensile strength is about a hundred times higher than that of steel [43]. However, some drawbacks have also been found, as they tend to cluster in bundles—thus making the interaction with the cement matrix inefficient—and the bond between CNTs and the matrix is weak [137].

**Figure 6:**
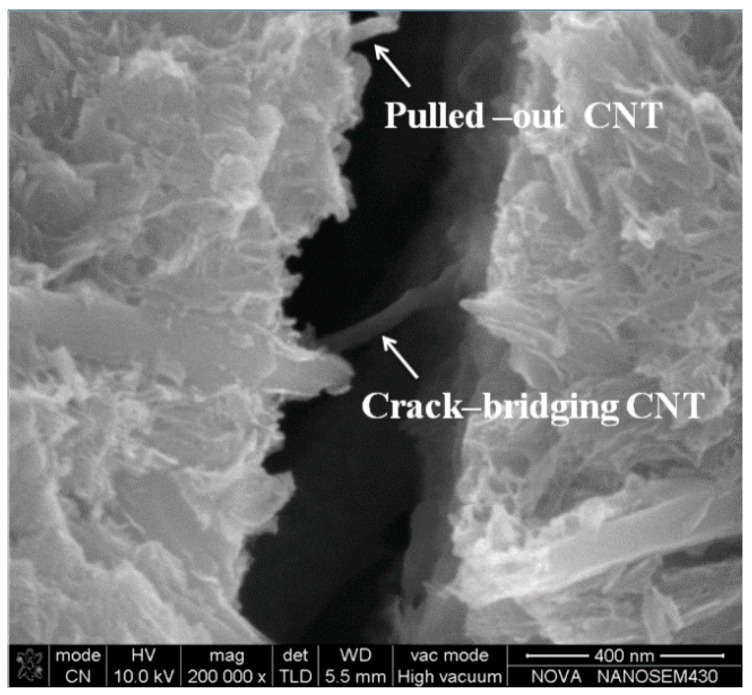
Crack bridging with MWCNTs (adapted from [141]).

In the context of mechanical improvements, CNTs are intended to move the reinforcing behaviour of carbon fibres from the macroscopic to the nanoscopic level [139]. In the mesoporous environment of concrete, these nanofilaments inhibit crack generation and arrest growth at the nanoscale. Besides, they act as fillers—making the C–S–H net to be denser—and enhance the quality of the cement paste-aggregate interface [140]. A micrograph of this feature is shown in Figure 6.

Considerable efforts to obtain CNT composites have already yielded positive results with different compounds. For instance, CNTs can be dispersed in an adequate solvent in the polymeric matrix, with or without functionalization. In metallic or ceramic matrices, mechanical methods can be used satisfactorily [43]. On the contrary, the cement matrix is not suitable for those approaches. The functionalization of CNTs can lead to changes in their chemical structure and affect the electrical behaviour of the nanoelement. The reason resides in the cleavage of the hybrid sp^2^ C═C bond, which leads to a decrease in the electrical conductivity [142]. Nevertheless, Fattah et al. [137] described how functionalization with the –COOH group increased the solubility and the bonding strength of CNTs in the cement matrix [54]. Isfahani et al. [143] did not find any improvement in the dispersion process when applying sonication to the CNT suspension in water and, then, adding it to the cement mortar. With the scope of adding CNTs to cement matrices, Stynoski et al. [144] enhanced the solubility of CNTs in water through their functionalization with nanosilica particles.

Regarding CNTs, researchers have focused on the dispersion methods that are compatible with the chemistry of cement paste. A common line of action consists in the use of superplasticisers as dispersing agents [57]. Compared to the effects of chemical modification (functionalization), Alkerabi et al. [133] observed a higher increase in compressive strength when applying physical modification (superplasticiser/surfactant). Siddique et al. [145] and Chuah et al. [31] published in 2014 their respective reviews on the studies dealing with the improvement in the fundamental mechanical properties of cement matrices, taking into account the synthesis method, the method of dispersion and the type of superplasticiser and surfactant used. More recent findings on the reinforcement of cement matrices are collected in Table 3.

Mohsen et al. [152] analysed the relation between the duration of the sonication applied to CNTs and the flexural strength achieved. By using a polycarboxylate-based superplasticiser, they achieved the following results. With 0.15 wt % of CNTs, the flexural strength practically stood still from Minute 15 to 60 of the sonication process. With 0.25 wt % of CNTs, the flexural strength increased linearly with sonication time. In their study on MWCNTs, Konsta-Gdoutos et al. [153] reported that, together with the application of ultrasonication, a weight ratio of surfactant to MWCNTs close to 0.4 was needed in order to achieve an optimum dispersion. Moreover, they found that long MWCNTs were more effective at reinforcing the cement matrix than short MWCNTs.

The nanosize of carbon allotropes makes them suitable to be combined with higher scaled inclusions, as it can be inferred from the presence of fly ash, silica fume, carbon nanofibers and PVA fibres in Table 3. Fly ash and silica fume are micro-sized pozzolanic materials used in the production of HPC. Regarding the pore-filling function, it can be observed that a gradation in the size—and cost—of the materials seems to be more efficient than the inclusions of different sizes used separately.

Thanks to their piezoresistive strain-sensing capabilities, CNTs are useful to monitor the structural health of a cement-matrix element [154]. The electric resistivity of a construction material can be used as a parameter of the stresses applied over the different structures [155,156]. The strain sensing capability can be reversible or irreversible. The detection of irreversible strains is a sign of health issues in a given structure. Reversible strains can be monitored in order to measure dynamic loads [157]. Typically, the sensing of reversible strains is more difficult because they are usually smaller than irreversible strains and it is a process that requires real time monitoring [158].

D’Alessandro et al. [159] experimented on the effects that this electrical feature could have on the cement matrix, paying special attention to the application of chemical dispersants and the use of different mixing strategies that did not bring down the electric conductivity of CNTs. Based on their results, it can be conclude that, despite the low cost of the electrical equipment involved, permanent monitoring of the structural stress might not be an interesting tool for all buildings—technically and economically speaking—but it may find an application in civil structures subjected to complex dynamic loads. The electrical resistivity of such nano-modified concrete also relies heavily on temperature [160] and, in connection to this point, Zuo et al. [161] suggested that this property could be applied to traffic pavements and structures in order to monitor traffic: number of vehicles, weight-in-motion measurement, vehicle speed and temperature sensing.

Another application of CNTs is linked to the electromagnetic shielding, which would protect both devices and human health [162,163]. Nevertheless, CNTs are not currently being used for this purpose on a global scale, as there are various low cost materials that are also able to perform this task. Alternatively, Khushnood et al. [164] proposed the use of carbonaceous nano/micro inerts, obtained from the carbonisation of agricultural waste, which are quite effective at enhancing the electromagnetic interference. Besides, they are highly cost effective and very efficient, as far as dispersion is concerned, when compared to CNTs. Another economical alternative to CNT-concrete can be achieved by using the CNT as a coating [165].

#### 4.2.2. Carbon Nanofibres

CNFs are cylindrical nanostructures with graphene layers that can be stacked according to three different patterns: in the shape of cups, cones, or plates. Their average diameter and length vary from 70 to 200 nm and 50 to 200 µm, respectively [166]. They have a tensile strength of circa 8 GPa, slightly inferior than that of CNTs, and their price is 50 times lower when compared to the cost of these nanofilaments [167]. The vapour deposition fabrication method enables the production of CNFs at such commercially viable prices [168].

Yazdani et al. [166] compared the reinforcement obtained in mortar with CNTs and with CNFs. With 0.1 wt % of CNTs, the compressive and flexural strengths attained an enhancement of 54% and 14%, respectively. The same weight-to-cement ratio of CNFs achieved an improvement of 68% and 8% with regard to the same parameters. This superiority of CNFs to improve the flexural strength was also reported by Danoglidis et al. [169]: an improvement of 106% and 87% with CNFs and CNTs, respectively. Metaxa et al. [170] demonstrated the effectiveness of CNFs in the cement paste, both on their own and together with PVA fibres. After a 28-day curing period, a 0.048 wt % dosage of CNFs increased the flexural strength and the toughness by 36.4% and 21.7%, respectively. A 0.54 wt % dosage of PVA fibres achieved an increase of 5.4% in the flexural strength and of 28 times in the toughness. When both fibres were used simultaneously, an improvement of 32.7% in the flexural strength was achieved, as well as an increase of 30 times in the toughness.

At 20 °C, the electrical conductivity of CNFs is 10^5^ S·m^−1^, whereas the value for CNTs ranges from 10^5^ to 10^7^ S·m^−1^ [171]—the value for copper is 6 × 10^7^ S·m^−1^ [172]. Given such high conductivity, Galao et al. [173] reported that a CNF content of 2% by weight of cement and a fixed voltage of 20 V were able to prevent the freezing of the concrete specimen (with dimensions 30 × 30 × 2 cm^3^).

Gomis et al. [174] studied this heating function on the cement paste with different carbonaceous materials: graphite powder (13 µm diameter and 130 µm length), carbon fibres (13 µm diameter and 3 mm length), CNFs (20–80 nm diameter and more than 30 µm length), and MWCNTs (aspect ratio greater than 150). They confirmed the economic and technical viability of using those carbon-based materials to provide concrete with self-deicing capabilities (which, however, are not enough to self-melt snow). Moreover, they remarked the paramount importance of moisture in concrete, as the conductivity and, therefore, the applicable power increased as it did the moisture.

Gao et al. [175] determined the optimum content ratio of CNFs to maximise the piezoresistive performance of concrete specimens. They used three different types of CNFs, produced through several synthesis methods, which yielded diverse values of electrical conductivity. Gao et al. pointed out the importance of an adequate dispersion of CNFs, as well as the existence of a threshold of fibre concentration beyond which the electrical resistance remained the same regardless of the variation of the strain. Sanchez [176] achieved an improvement regarding the dispersion in the cement paste by using nitric acid as a surfactant.

Mo et al. [157] studied the strain sensing of self-compacting concrete by adding CNFs. They concluded that this nano-modified concrete was suitable to work as a permanent strain sensor, which would help to detect damage in the structure. However, the correspondence between the loads and deformations was not precise enough to safely assume that this concrete could be used as a reversible strain sensor.

#### 4.2.3. Graphene Oxide

Graphene oxide consists of graphite that has been oxidised to intersperse the carbon layers with oxygen molecules and, then, has been reduced to separate the carbon layers completely into individual or few-layer graphene [177]. The carboxyl, hydroxyl and epoxy functional groups confer high processability and water-solubility to GO but, conversely, efface the excellent electrical properties of graphene and degrade its mechanical properties [178], as it can be observed in Table 3. Nevertheless, it stands out as a reinforcement that can compete economically with the extensively studied CNTs for the following reasons: GO is easier to produce, possesses higher solubility in the aqueous cement matrix, and its sheets increase the nucleation area available for the C–S–H gel [179]. In short, the functional groups and the high interface area provide GO with higher reactivity.

Such advantages are reflected in the mechanical enhancement achieved. A 0.05 wt % of GO increases the compressive strength of the cement paste by 15%–33%, and the flexural strength by 41%–59% [180]. GO serves as a nucleation agent in cement hydration reactions, stimulating the growth of the C–S–H gel [31]. Moreover, the cement paste containing GO shows a ductile behaviour. Such high efficiency makes GO an interesting object of study as a nanoreinforcer, as Table 3 shows. Lu et al. [34] made a review in which they compared the mechanical performance of GO, CNTs and CNFs, as well as the electrical performance of these last two elements.

#### 4.2.4. Pristine Graphene and Graphite Nanoplatelets

Graphene is, currently, the strongest known material. As it can be deduced from Table 2, its tensile strength is approximately 100 to 300 times higher than that of steel. Therefore, it constitutes an attractive line of research in the construction field. Additionally, special properties such as elasticity [181], excellent thermal characteristics [182] and electrical conductivity [183] provide the cement matrix with several smart functions [31]. However, and because of this unique behaviour, the research focused on graphene has been driven by applications in the field of electronics [184], nanofiltration [185], or biocompatible devices [186]. The term biocompatibility means that a device can operate inside a body without causing any adverse reactions [187].

Pristine graphene is not suitable to be combined with the cement matrix. Although it is true that it is an expensive material, the main reason underlying this unsuitability is the highly hydrophobic behaviour of graphene: it has no appreciable solubility in most solvents [188]. Therefore, cement-based composites reinforced with carbon-based nanomaterials are overshadowed by CNTs and GO. To date, graphene has been successfully implemented in the cement matrix in the form of nanoplatelets, which consist of layers with a thickness less than 100 nm and a diameter of several micrometres. In scientific literature, these elements are often named graphite nanoplatelets (GNPs) [146,189].

Meng et al. [146] further extended the tensile resistance of UHPC by using GNPs and carbon nanofibres. UHPCs are based on an optimised gradation of their granular constituents, a water-to-cementitious materials ratio lower than 0.25 and a high percentage of discontinuous steel fibre reinforcement. They reach a compressive strength over 150 MPa and, because of their closely packed structure, their durability is significantly higher when compared to HPC (High Performance Concrete) [190]. Meng et al. noticed that while compressive strength rose by 4.5%, nanomaterials significantly contributed to arrest crack generation: with 0.3 wt % of GNP, tensile strength and energy absorption increased by 45% and 153%, respectively. With 0.3 wt % of CNF, the same parameters increased by 56% and 108%, respectively. The fundamental details of the mix and the procedure are shown in Table 3. Another recent study carried out by Wang et al. [191] yielded the following results: with 0.05 wt % of GNPs, the flexural strength of cement paste specimens increased by 16.8% after a 28-day curing period.

Once a structure is damaged and presents cracks, they can be filled with CaCO_3_ precipitated by bacterial activity, either by embedding bacteria in the blend or by being applied as a coating. In this context, nanomaterials have also been used to encapsulate the bacteria. Khaliq et al. [192], for example, after encapsulating the *Bacillus subtilis* in GNPs and adding it to the blend, were able to fill 0.81 mm wide cracks. After the cement matrix was hardened and the cracks were induced, calcium lactate was applied as an organic precursor and the expansive precipitation of calcium carbonate from the bacterial activity filled the cracks. Seifan et al. [193] provided an overview on different microbial approaches to induce the precipitation of CaCO_3_ inside the cement matrix. In their study, it was observed that the materials and techniques which are compatible with the bacteria primarily depended on their metabolic pathways: autotrophic—when microbes react to CO_2_—and heterotrophic—when the bacteria react to organic compounds. Kim et al. [194] spotted differences in the amount of precipitation between five bacteria, thus finding three that produced a higher quantity than the *S. pasteurii*—one of the most commonly used. Apart from studying the bacterial activity, Muhammad et al. [195] made a review on the studies dealing with self-healing concrete. They concluded that, apart from bacterial activity, the use of polymeric and supplementary cementitious materials were the most common practices.

#### 4.2.5. Carbon Black (CB) Nanoparticles

CB, which is produced by an incomplete combustion, is essentially composed of carbon atoms in the form of an amorphous molecular structure. More in particular, it is a structure of crystalline arrays of condensed rings. Since these arrays are randomly oriented, they have some open edges with unsatisfied carbon bonds, what implies chemical reactivity [196].

Wen et al. [197] compared the electrical characteristics that carbon fibres and CB provide to the cement paste: with the same content ratio, carbon fibres were more effective than CB at increasing the electrical conductivity of the cement matrix and at shielding electromagnetic interferences. A partial replacement of up to 50% of the carbon fibres for CB maintained the conductivity at a lower cost, and, therefore, it facilitated the use of this composite in deicing, electrical grounding, and cathodic protection applications.

The reduced size of CB and its electrical conductivity make it an economical method for protecting steel rebars from corrosion. Masadeh [198] specifically studied this feature of concrete containing CB. In the experiment conducted by this author, concrete specimens were left in a tank with a 3.5% NaCl solution for 6 months. The subsequent analysis of the specimens concluded that corrosion decreased as the CB content increased. With a weight-to-cement ratio equal to or higher than 0.4%, the chloride permeability of the specimens was reported to be very low.

**Figure 7:**
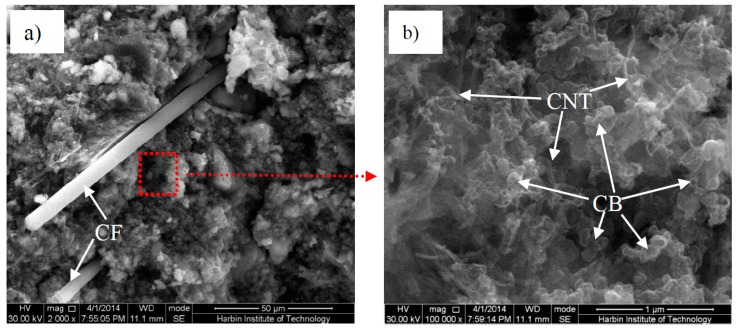
SEM image of a triple carbon-modified cement matrix: (**a**) Carbon fibres at 2000 magnification; and (**b**) CNTs and CB at 100,000 magnification (adapted from [199]).

Qiao et al. [199] studied triple-scaled carbon-inclusions in concrete. The objective was to use the combination of carbon fibres, CNTs and CB as an anode for the impressed current cathodic protection in reinforced concrete structures. The optimum dosages in the weight-to-cement ratio resulted to be 3% for carbon fibres, 1.5% for CNTs and 2% for CB. This mix achieved a good service life under an extreme polarization potential and it was immune to chloride attack. These three inclusions are illustrated in Figure 7.

Despite being less effective than carbon fibres [197], Xiao et al. [200] reported a good self-sensing behaviour of the cement matrices containing CB: an increase in the compressive stress led to a linear decrease in the fractional change of the electrical resistance [175]. Monteiro et al. [201] measured both the mechanical and the piezoresistive performances of mortar specimens. They found that the additions of CB with a content of approximately 4% w/b (weight-to-binder ratio) were favourable to improve the compressive and tensile strengths, while the optimum for the best piezoresistive performance fell within the range of 7%–10% w/b. Chung [202] described the differences derived from the use of CB, CNFs and GNPs with regard to the electromagnetic performance of the cement matrix.

## 5. Health impact of Nanomaterials

The on-going growing industry of nanoproducts has led to an increase in the studies about the effects that nanomaterials have on the environment and human health [203,204]. More specifically, there has been an increase in the number of papers that review the health and safety considerations related to the use of nanomaterials in the construction industry during their whole life cycle [205,206]. The main reasons for their toxicity lie in their reduced size and high reactivity.

Many studies have proven the harmful effects of airborne particulates on the respiratory and cardiovascular systems, including a greater incidence of atherosclerosis and a higher rate of asthma [207]. Well known examples of these particulates—which have a diameter of less than 100 nm—are usually generated by high temperature processes, such as welding and smelting, combustion, and industrial processes [208]. Now, nanomaterials are being manufactured on a large scale, and, therefore, the risks for workers and users should be assessed.

Nanosilica particles with a 42 nm diameter were demonstrated to penetrate into the human skin [209]. Hirai et al. [210] reported that nanosilica with a 70 nm diameter, applied for three days on mice skin, could penetrate and be transported throughout the body via the lymphatic system [211].

The second most used nano-sized metal oxide worldwide is TiO_2_ [212]. Chang et al. [213] analysed 62 papers which focused on the study of the health consequences from the exposure to nano-TiO_2_. In these pieces of research, some clues towards the hypothesis that this nanomaterial could have an impact on human health were found. Nano-TiO_2_ was detained in several organs and possible cell damage was reported. However, further research is still needed to demonstrate its toxicity for the human body, especially epidemiological studies, as they can show the relation between the occupational exposure and the development of health problems more directly.

Ong et al. [214] reviewed the studies on SWCNTs, from their absorption into a body to the accumulation and induction of organ-specific toxicity. Although earlier studies had reported that the harmful effects of SWCNTs were similar to those of other conventional fibres such as asbestos, recent pieces of research have suggested that the nanometric nature of SWCNTs can have further consequences on human health. For example, Ong et al. indicated more toxic effects on numerous cell types, when compared to the same nanoparticulate mass for carbon and quartz—which are commonly adopted as yardsticks for harmful particles. Both SWCNTs and MWCNTs pose risks to the respiratory system [215] and exhibit antibacterial properties [205]. Singh [216] reviewed the toxicological studies on the graphene family nanomaterials in the context of their applications.

A common conclusion of the medical studies regarding health-related issues of nanomaterials is the need of further research to confirm these risks to human health. To this end, many researchers have remarked the convenience of standardising the nanomaterial itself [214,217], as well as the protocols of both the experiments and long-term studies [218,219].

## 6. Discussion of Results

The studies included in this paper demonstrate that nanotechnology applied to the cement matrix is still in an intense phase of research: searching for more efficient and environmentally sustainable synthesis methods, exploring attractive applications, and designing the first prototypes of nano-technological buildings. Table 4 and Table 5 gather the findings and tested applications included in the analysed studies.

Many of these pieces of research share a common characteristic: a significant variability in the strength parameters when reinforcing cement matrices. The reasons for this variation lie in all the stages of the research process. Firstly, there is not a common and widely accepted synthesis method for nanomaterials and research groups have to use expensive devices to identify the nanostructure of the material used. Nanoparticles are in a favourable situation compared to carbon-based nanomaterials, as their manufacture is easier and has been evolving for a longer time. However, in cement products with nanoparticles, the proportions of the mixture and the components are far from being commonly adopted in the short term. Secondly, the characteristics and preparation of specimens follow different standards, as it happens to testing devices. The properties of the specimens will depend on the instruments and materials available where the experiment takes place. Thirdly, there is no coincidence in the mechanical parameters measured in each study. Compressive strength is usually provided, but flexural, toughness, and tensile tests are carried out less frequently.

Nanoparticles have been proven to provide valuable improvements in the mechanical properties of the cement matrix, as well as new advanced properties—such as strain and temperature auto-sensing and self-cleaning capability. Nevertheless, it is a fact that laboratory work at the molecular level is a costly activity and the companies from the cement and construction sectors usually work with limited budgets. Expensive microscopes are needed to examine nanostructures, high technology is required to synthetise high purity graphene and CNTs [220], and an extra effort is also necessary to disperse these nanoinclusions uniformly within the cement matrix. Consequently, both the commercial nanoinclusions currently available and the products made of them are still limited [221]. Mahdavinejad et al. [222] highlighted the lack of coherence between the academic field and the industrial requirements.

Experimentation with GO- and CNT-cement is a very active line of study. The obstacles derived from their high cost and poor binding properties are expected to be gradually overcome in the future. At present, further work regarding building Codes needs to be done in order to achieve a widespread application of these nano-modified composites and, even of the well-known macroscopic fibre concretes [54]. In cement composites, the research on CNTs prevails over the studies on CNFs. However, CNFs, due to their lower cost, are still an interesting object of study with regard to the reinforcing and electrical functions. For the same reason, CB is used as a nanomaterial in order to enhance the conductivity of the cement matrix.

As previously exposed, additional physical properties—beyond the fundamental mechanical requirements for the cement matrix—have already been successfully tested. The study of smart structures has been highlighted through the examples of the strain self-sensing and auto-healing capacities. However, there is still a lack of on site experiments and prototypes that include such features.

Medical studies on the health risks posed by nanomaterials have been warning about absorption, detaining or cell damage in animals. These findings constitute a strong indicator of the possible risks to human health. Further research is strongly recommended to confirm this hypothesis.

Given the limited international commitment towards the introduction of multi-functional concrete in architecture, influential building certificates—such as BREEAM and LEED—can play a key role to foster innovation within construction. In this regard, a remarkable example would be the last guide on the selection of materials published by The Concrete Centre and developed within the REEAM framework [223], as it opens the door to provide new features to concrete with regard to the reduction of gas emissions, the comfort of the user, the use of recycled materials, etc.

## 7. Conclusions

Although most of the activity around nanoinclusions is still in a research stage, some findings have already proven that there is great room for improvement in the mechanical performance of the cement matrix, either with the addition of nanoparticles or with the use of carbon-based nanomaterials. Moreover, nanoelements can usually be combined with micro- and macro-admixtures and reinforcements in order to further enhance the strength of the hardened cement composite. Nevertheless, the variability in the strength parameters of cement-matrix composites reflects the need of standardising the activities related to nanotechnology.

As previously mentioned, it is important to channel efforts in order to provide efficient and, therefore, standardised synthesis methods for nanomaterials to improve their production on a large scale. If research groups had access to similar products, the comparison of results would be more reliable. The next step would be to make standards based on the most efficient processes for mixing the cement-based composite blend.

Standardisation is a key line of action, as high quality standards are needed to facilitate the transferability of the results from the research field to the global market. Nanoscience is a new promising field that, in order to develop further, needs to establish an internationally agreed terminology, as well as commonly adopted methods of measurement and characterisation. Construction would particularly benefit from the standardisation of technical requirements, since its activity is regulated by mandatory codes that evolve, in the long term, on the basis of solid empirical experience.

Nanotechnology also helps to decrease the environmental impact derived from the construction activity. Firstly, the strengthening provided by nanoproducts can lead to a reduction in the carbon footprint of the cement matrix by cutting down the consumption of cement. Secondly, recycled elements have been successfully tested as nanoadmixtures in the cement matrix, or, at least, the reinforcing effect of nanomaterials provides room to include recycled elements—which would otherwise imply an expensive disposal process. Influential building certificates, such as LEED and BREEAM, will probably play a key role in fostering the application of innovative technologies within the construction industry.

Several novelty properties of nanomaterials, which are primarily based on the electrical characteristics of nanoelements, have been successfully tested in lab work. A common research topic is the piezoresistive characteristic of CNTs, which could lead to the design of a load-sensing structure or a thermal-sensing coating. These advanced features can be used by entrepreneurs as a way to differentiate their products from those that have traditionally been in the market.

Research groups and private initiatives should carefully choose the line of research they are eager to follow, given the high variability of results found in this field. Regardless of this aspect, it cannot be denied that, taking into account the huge consumption of Portland cement worldwide, any interesting improvement in the cement matrix that reaches an acceptable cost-effectiveness ratio will have great economic and positive environmental impacts at an international level.

## Figures and Tables

**Table 1:** Findings on the enhancement of the mechanical properties of cement-matrix composites.

Weight-to-Cement Ratio of Inclusion	Type of Cement-Matrix Composite ^1^	Dispersion Technique	Increase in Performance (%) ^2^	References
1% NS ^3^	Concrete	Mixer	12.96% Compressive Str. ^4^	[77]
2% NS	Mortar	Ultrasonication	48.7% Compressive Str.; 16.0% Flexural Str.	[78]
2% NS	Cement Paste; superplasticiser; Quartz Aggregate;	Mixer	8.0% Compressive Str.; 37.5% Tensile Str.	[79]
2% NS, 10% SF	Cement paste; Superplasticiser; Quartz aggregate;	Mixer	6.0% Compressive Str.; 19.4% Tensile Str.	[79]
6% NS	Mortar; Superplasticiser	Mixer	142% Compressive Str.	[80]
5% Nano-MK	Mortar; Superplasticiser	Mixer	28.0% Compressive Str.	[81]
0.6% Nanoclay	Cement Paste	(Not Specified)	13.24% Compressive str.	[25]
10% NS; 18% Nanoclay;	Mortar; Superplasticiser	(Not Specified)	201.7% Compressive Str.; 413.8% Tensile Str.	[82]
1% Nano-MgO	Mortar	Ultrasonication; Superplasticiser; Surfactant	80% Compressive Str.; 70% Flexural Str.	[83]

^1^ Unless otherwise specified, ordinary Portland cement (OPC) ASTM (American Society for Testing and Materials) type I was used; ^2^ Specimens cured for 28 days; ^3^ Nanosilica; ^4^ Strength.

**Table 2:** Mechanical properties of graphene, CNTs and GO (adapted from [31]).

Material	Elastic Modulus (GPa)	Tensile Strength (GPa)	Elongation at Break (%)	Diameter/Thickness (nm)	Aspect Ratio	References
Graphene	1000	130	0.8	~0.08	6000–600,000	[116,117]
CNTs	950	11–63	12	15–40	1000–10,000	[118,119]
GO	23–42	~13	0.6	~0.67	1500–45,000	[120,121]

**Table 3:** Recent results on the improvement in the mechanical properties of cement matrices to date.

Weight-to-Cement Ratio of Nanoinclusion ^1^	Type of Cement-Matrix Composite ^2^	Dispersion Technique	Increase Referred to OPC Specimen (%) ^3^	References
0.3% GNPs; 40% FA; 5% SF	UHPC ^4^; Portland ASTM Type III	Bath Sonication; Polycarboxylate Superplasticiser	45% Tensile Str.; 153% Energy Absorption	[146]
0.3% CNFs; 40% FA; 5% SF	UHPC; Portland ASTM Type III	Bath Sonication; Polycarboxylate Superplasticiser	56% Tensile Str.; 108% Energy Absorption	[146]
1% CNTs	Mortar	CNT–COOH	40% Compressive Str.	[137]
0.15% CNTs; 30% SF	Mortar	CNT–COOH; CNT–OH ^5^	20% Compressive Str.; 50% Flexural Str.	[147]
1.5% GO	Pavement Concrete	Polycarboxylate Superplasticiser	48% Tensile Str.	[148]
0.05% GO	Cement Paste	Polycarboxylate Superplasticiser	40.4% Compressive Str.; 90.5% Flexural Str.	[149]
0.05% GO	Mortar	Superplasticiser	24.4% Compressive Str.; 70.5% Flexural Str.	[149]
0.08% GO; 80% FA; 2% SF; 2% vol. PVA fibres	Mortar	Superplasticiser	24.8% Compressive Str.; 37.7% Tensile Str.; 80.6% Flexural Str.	[150]
1% GO	Mortar	Polycarboxylate Superplasticiser	86.3% Compressive Str.	[151]

^1^ GNPs: Graphite nanoplatelets, FA: Fly ash, SF: Silica fume, CNFs: Carbon nanofibres; ^2^ Unless otherwise specified, ordinary Portland cement ASTM Type I was used; ^3^ Specimens cured for 28 days; ^4^ Ultra high performance concrete; ^5^ Both functionalizations yielded similar results. Specimens cured for 14 days.

**Table 4:** Most remarkable properties enhanced or provided by nanoparticles.

Nanoinclusion	Compressive Str. and Pore-Filling	Flexural Str.	Freeze/thaw Cycles Resist.	Steel–Matrix Bond	Hydration Accelerator	Hydration Retarder	Shrinkage Reducer	Photocatalytic	Hydrophilic Coatings	Hydrophobic Coatings
Nano-SiO_2_	●	●	●	●	●	-	-	-	-	●
Nano-Al_2_O_3_	●	●	-	●	-	●	-	-	-	-
Nano-Fe_2_O_3_	●	●	-	-	-	-	-	-	-	-
Nano-CaCO_3_	●	●	-	-	●	-	●	-	-	-
Nano-MK	●	●	-	-	-	-	-	-	-	-
Nanoclay	●	●	-	-	-	-	-	-	-	-
Waste glass Nanoparticles	●	●	-	-	-	-	-	-	-	-
Nano-MgO	●	●	-	-	-	-	●	-	-	-
Nano-TiO_2_	●	●	-	-	-	-	-	●	●	-
Nano-TiO_2-*x*_N*_y_*	●	●	-	-	-	-	-	●	-	-

**Table 5:** Most remarkable properties enhanced or provided by carbon-based nanomaterials.

Nanoinclusion	Compressive Str. and Pore-Filling	Flexural Str.	Strain-Sensing	Encapsulating of Bacteria for Healing Capability	Thermal Sensing	Electromagnetic Interferences Shielding	Electrical Heating	Cathodic Protection for Steel Elements
GNPs	●	●	●	●	-	-	-	-
CNTs	●	●	●	-	●	●	●	●
CNFs	●	●	●	-	-	-	●	-
CB	-	-	●	-	-	●	●	●
GO	●	●	-	-	-	-	-	-
